# Study of the Properties of CdS:Al (R = [Al^3+^]/[Cd^2+^] = 0.30, 0.40, 0.50) Thin Films Grown by the CBD Method in an Ammonia-Free System

**DOI:** 10.3390/molecules28083626

**Published:** 2023-04-21

**Authors:** Raju Prasanna-Kumari, Daniela Herrera-Molina, Arturo Fernández-Pérez, Jesús E. Diosa, Edgar Mosquera-Vargas

**Affiliations:** 1 Grupo de Transiciones de Fase y Materiales Funcionales, Departamento de Física, Universidad del Valle, Santiago de Cali 760032, Colombia; 2 Departamento de Física, Facultad de Ciencias, Universidad del Bio-Bio, Collao 1202, Concepción 4030000, Chile; 3 Centro de Excelencia en Nuevos Materiales (CENM), Universidad del Valle, Santiago de Cali 760032, Colombia

**Keywords:** thin film, CBD method, structural properties, optical properties

## Abstract

CdS:Al thin films were fabricated on a glass substrate using the CBD method. The effect of aluminum incorporation on the structural, morphological, vibrational, and optical properties of CdS thin layers was investigated by X-ray diffraction (XRD), Raman spectroscopy (RS), atomic force microscopy (AFM), scanning electron microscopy (SEM), and UV-visible (UV-vis) and photoluminescence (PL) spectroscopies. XRD analysis of deposited thin films confirmed a hexagonal structure with a preferred (002) orientation in all samples. The crystallite size and surface morphology of the films are modified with aluminum content. Raman spectra exhibit fundamental longitudinal optical (LO) vibrational modes and their overtones. Optical properties were studied for each thin film. Here, it was observed that the optical properties of thin films are affected by the incorporation of aluminum into the CdS structure.

## 1. Introduction

The development of semiconducting nanomaterials doped with metal ions has received much interest from different research groups because their properties differ from those of bulk materials [1,2,3,4]. Among II–VI semiconductor nanomaterials, cadmium sulfide (CdS), an n-type semiconductor, is of interest in photovoltaic solar cells due to its bulk direct energy gap (*E_g_*) of 2.42 eV at room temperature (RT) [1,5,6,7,8]. In addition, CdS is a promising and interesting material for nanodevice applications due to its physicochemical properties [3,9,10]. Previous works reported that various synthesis methods have been used to grow CdS thin films [2,3,4,5,9,10,11,12,13], where chemical bath deposition (CBD) is widely employed for its scalability, low cost, and easy manufacturing process. This technique has been used for several years and is based on the deposition of metal chalcogenide semiconductor thin films on a solid substrate from a chemical reaction occurring in an aqueous solution [14,15,16,17]. This precursor solution contains a source of metallic cations, one or more complexing agents, and a source of anions. This chemical reaction is controlled in such a way that the rate of formation of semiconductors in the solution is slow enough to allow the growth of a thin film over the substrate, avoiding precipitation [16,18]. Additionally, CBD does not require high vacuum systems and/or high temperatures, unlike other methods such as RF-sputtering deposition [19] or deposition by evaporation [20]. In addition to being environmentally friendly [2,21], the CBD method exhibits good reproducibility for the mass production of thin films on an industrial scale [22]. 

It is very important to note that previous reports on CdS deposition with the CBD method use ammonia in a chemical bath as the complexing agent and hydroxide source [23]. Therefore, it is necessary to avoid the use of ammonia if we want to use this manufacturing method on an industrial scale since it is very harmful to the environment and human health. In this sense, it has been reported that the physical properties of CdS films manufactured by CBD are strongly dependent on the complexing agent used in their fabrication [24,25]. For this reason, other authors show that good-quality CdS thin films can be fabricated by CBD without ammonia; for example, using other complexing agents such as sodium citrate [5,7] and nitrilotriacetic acid [26].

On the other hand, the manufacturing of doped CdS films using CBD with atoms of group III, such as Al [1,5,6,7,8,11,13,27,28], In [29], and Ga [30], allows us to modify their properties with respect to the undoped CdS film. This is because group III metals rapidly diffuse impurities in the structure of this semiconductor, causing important changes in the physical properties (structural, morphological, optical, and electrical) of CdS [31]. For example, both increases and decreases in the CdS bandgap have been reported with Al doping, which is related to the reagents used in the chemical synthesis, the surface morphology of the films, and the deposition times [8,11,13,32]. However, the substitutional and/or interstitial replacement of Cd^2+^ ions in the lattice by Al^3+^ ions depends on the dopant concentration in the solution; therefore, the modification of the crystallite size, lattice strain, and interplanar distance is related to the dopant content in the CdS film [7,32]. In this sense, the range of optical, structural, and morphological magnitudes that can be obtained in CdS makes it optimal for their applications in optoelectronic devices [11,13,23,33,34]. Furthermore, it is important to note that ammonia is one of the main reagents used in the synthesis of thin films using the chemical bath deposition method; however, it is unfavorable from a green chemistry perspective, as the synthesis processes are toxic and the energetic reactions exhibit high consumption. Additionally, due to highly toxic reactions, exposures are limited as much as possible and use the least amount of toxic material possible. Low-temperature synthesis is required to form thin films, which could result in significant energy savings, particularly when considering a scalable method of synthesis. 

Currently, Al is interesting as an effective dopant that increases the concentration of carriers in CdS, thus decreasing its electrical resistivity [7,32] and modifying its charge neutrality level on a Schottky-contact CdS/Ag [5]. Likewise, Al is an interesting dopant due to its lower ionic radius than other dopants and its low cost, which make it ideal for the generation of window-type materials [16] and rectifying contacts [5,35]. In this sense, this type of film and the method of synthesis using ammonia-free systems are of great interest to manufacturers of optoelectronic devices based on this semiconductor [9,36]. Therefore, here, we study the incorporation of aluminum atoms as dopants (with a molar ratio in the solution R = [Al^3+^]/[Cd^2+^], R = 0.30, 0.40, 0.50) in CdS thin films grown by the CBD method in an ammonia-free system. However, we used some previously reported cadmium sulfide figures from Ref. [1] to complement and support the discussion of the manuscript and the study. In addition, the effects of doping on the properties of CdS thin films are investigated in detail using different characterization techniques. The structural characterization was carried out using X-ray diffraction (XRD) and Raman spectroscopy (RS), along with morphological characterization by atomic force microscopy (AFM). In addition, AFM was used to determine the film thickness and surface roughness. The optical properties of all deposited thin films were studied using ultraviolet-visible (UV-Vis) absorption and photoluminescence (PL) spectroscopy.

## 2. Results and Discussion

### 2.1. Morphological and Crystalline Characterization

**Figure 1:**
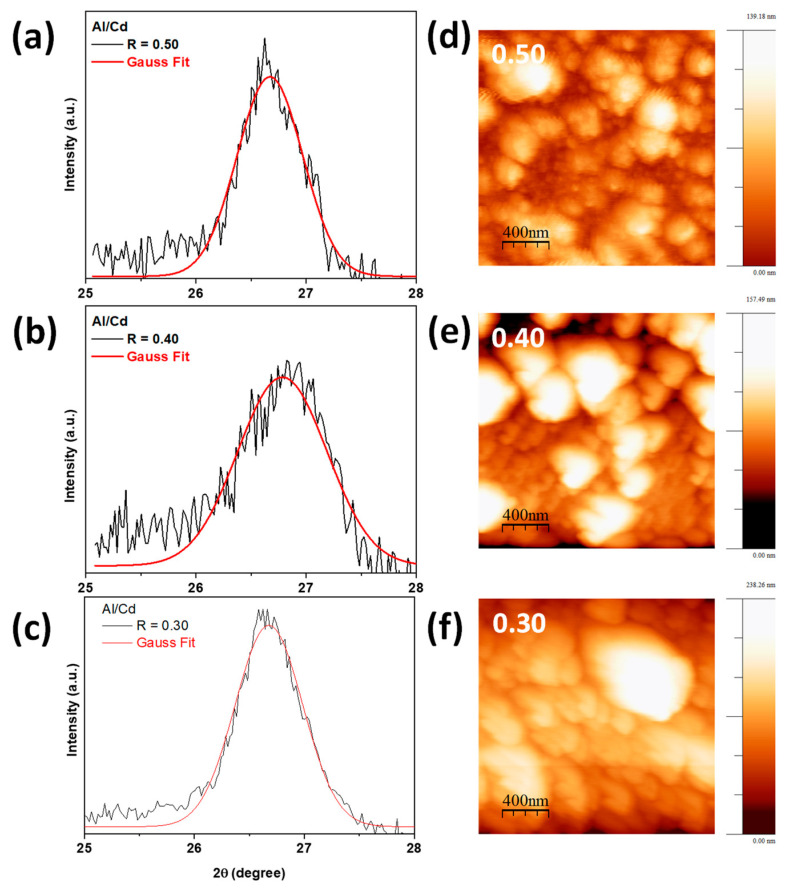
(**a**–**c**) (002) XRD plane and Gaussian fit, and (**d**–**f**) 2D AFM images of thin films grown at R = [Al^3+^]/[Cd^2+^] = 0.30, 0.40, 0.50 ratio.

Figure S1 and Figure 1a–c display the XRD patterns of the studied CdS:Al thin films grown using the chemical bath deposition (CBD) method. The measurements were carried out to determine the crystalline phase and structure in the thin films. For all samples, the diffractograms exhibit a characteristic main peak of around 2θ = 26.60° ± 0.02°, corresponding to a hexagonal wurtzite structure (PDF card no. 80-0006 [5] and no. 41-1049 [2]). The intense XRD peak corresponds to the preferential orientation of the growth of the plane (002), which indicates that the films were grown along the *c* crystallographic axis. In addition, there was no presence of aluminum phases or any binary phase, indicating that Al^3+^ ions do not change the crystalline structure of the CdS. For all samples, the crystallite size (*D_hkl_*) was determined from the line-broadening β (full width at half-maximum, FWHM) of the (002) diffraction peak and using Scherrer’s formula, $D_{hkl}=\frac{\kappa \lambda }{\beta cos\theta }$ [37]. Here, *κ* is a shape factor which is usually taken as 0.9 for spherical grains, λ is the X-ray wavelength (see Section 3.3.1), and θ is Bragg’s angle between the X-ray and the scattering plane. Therefore, the (002) plane has been fitted by a Gaussian function to obtain the 2θ and β values of the thin films (see Figure 1a–c). In this sense, the average crystallite sizes of all thin films have been estimated to be in the 11–14 nm range with Al content in CdS. Additionally, the lattice strain, ε, of the films was calculated using $\varepsilon =\frac{\beta cos\theta }{4}$. The results are reported in Table 1 and Figure S2. Therefore, the size ratio of the ionic radii of Al^3+^ (0.53 Å) and Cd^2+^ (0.95 Å) leads to a decrease in the average size of the crystallite, D, which is related to an increase in the strain, ε, of the thin films [26] (see Table 1). Thus, an increase in lattice strain in doped films could have an effect on their optical properties, which can be related to the substitutional replacement of cadmium (Cd^2+^) ions in the lattice with aluminum (Al^3+^) ions [1]. Comparing the results with those previously reported by Khallaf et al. [32], and recently by Willars-Rodríguez et al. [11], it was observed that the incorporation of aluminum (Al^3+^) ions in cadmium sulfide (CdS) thin films presents a decrease in the maximum X-ray intensity attributed to the preferential *c* orientation.

Figure 1d–f presents 2D AFM images of the thin films at the nanoscale level. AFM analysis also allows us to better study and understand the films; therefore, the morphology of the film was observed as well as the surface roughness. The RMS surface roughnesses for R = 0.30, 0.40, and 0.50 films were 41.2 nm, 25.2 nm, and 22.6 nm, respectively. In this sense, it is observed that a higher Al content of the films decreases their surface roughness, which is an important feature when metals are deposited on the semiconductor surface because it is related to the serial resistance of the metal contact and other parameters of the metal–semiconductor junction [5,35]. Furthermore, from the observed results, it can be concluded that the size of the crystallites decreased in the cadmium sulfide thin films with respect to the aluminum (Al^3+^) ions incorporated as dopants.

**Figure 2:**
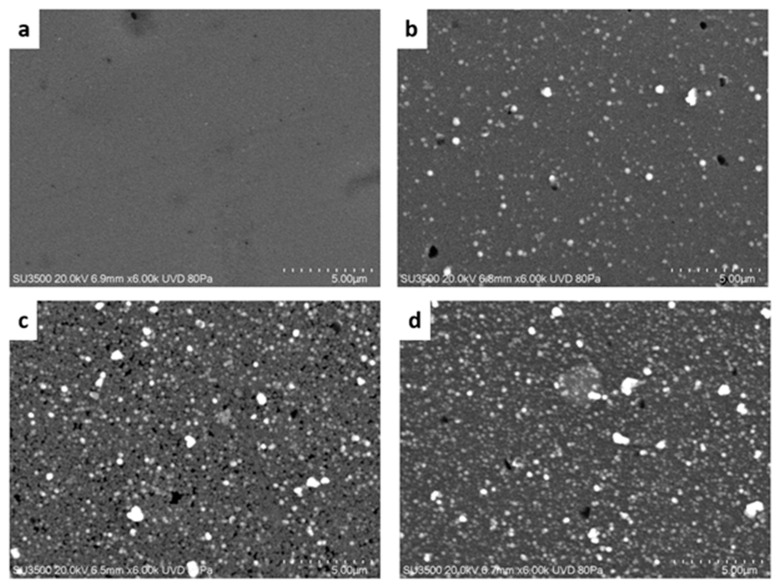
(**a**–**d**) SEM micrographs of the thin films grown at R = [Al^3+^]/[Cd^2+^] = 0.00, 0.30, 0.40, 0.50 ratio. An increase in the number of surface grains was observed with higher aluminum doping.

Regarding this, SEM images are presented in Figure 2a–d. For the undoped thin film (R = 0.00), its surface morphology is homogeneous and does not have grains on its surface. On the other hand, for the CdS:Al spherical surface, grains are observed and it is found that the number of grains increases along with the Al content, where the aggregates clump together to form grains of greater dimensions. In the case of the R = 0.30 film, a smaller amount of surface grains is observed, which is related to its optical characteristics, as will be shown in the next section. The average sizes of the grains on the surface of the glass substrate are estimated to be between 200 and 400 nm, respectively. However, for undoped thin films, these large grains are not observed, as was reported previously [1,11,32,38].

### 2.2. Spectroscopy Studies

Vibrational spectroscopy is a non-destructive characterization technique that provides information about the structure of the films. The Raman spectra are sensitive to the crystal quality, structural defects, and disorder of the synthesized samples. Here, Raman spectroscopy was carried out to study the influence of Al doping on the CdS structure. Therefore, Raman spectra were recorded using a 532 nm wavelength laser. With a hexagonal wurtzite structure, CdS belongs to the $C_{6v}^{4}$ (P6_3_mc) space group, and according to the group theory, single-crystalline CdS has nine sets of optical phonon modes (three longitudinal-optical (LO) and six transverse-optical (TO) modes) and three acoustic phonon modes (one longitudinal-acoustic (LA) and two transverse-acoustic (TA) modes) at the Γ-point of the Brillouin zone, classified as $A_{1}+2B_{1}+E_{1}+2E_{2}$ modes, where $A_{1}$, $E_{1}$, $E_{2}$ are Raman active and $B_{1}$ is Raman inactive [39,40]. However, due to both ionic–covalent forces in CdS, the $A_{1}$ and $E_{1}$ polar phonon modes split into longitudinal-optical (LO) and transverse-optical (TO) phonon vibrations. Moreover, the non-polar phonon mode, $E_{2}$, could be assigned to the $E_{2}$ (high) and $E_{2}$ (low) vibrational modes, respectively.

**Figure 3:**
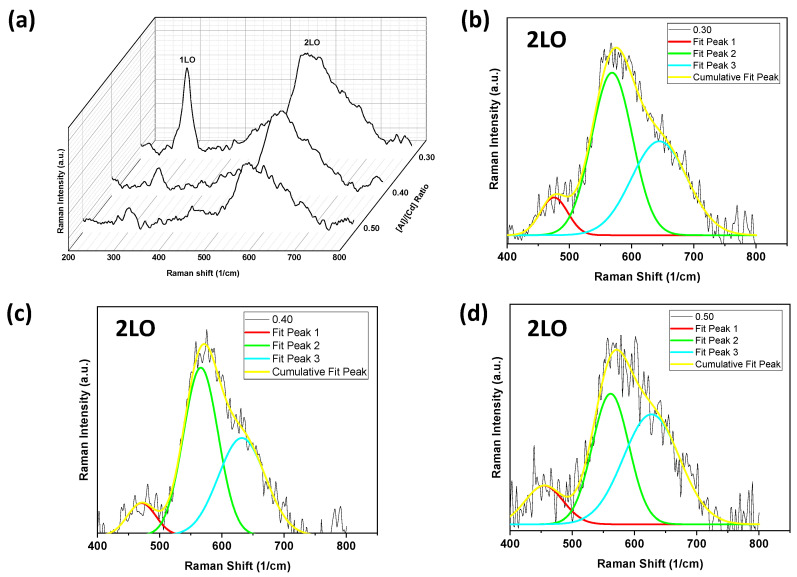
(**a**) Raman spectra of CdS:Al thin films. (**b**–**d**) Fundamental 2LO overtone fitted using a GaussAmp function.

The Raman spectra of the thin films show good crystallinity and are reported in Figure 3. Figure 3a–d show the characteristic peak centered at ~303 cm^–1^ corresponding to the longitudinal-optical ($A_{1}$ (1LO)) phonon and their overtones around 600 cm^–1^ ($A_{1}$ (2LO), Figure 3b–d). The peak at around 303 cm^–1^ is shifted by 1 cm^–1^ compared to the CdS reported in Ref. [1]. This shift could confirm the doping of aluminum in CdS. Additionally, it is observed that the intensity of the $A_{1}$ (2LO) multiphonon Raman scattering increases with respect to the $A_{1}$ (1LO) phonon, which decreases with the aluminum content. Here, the vibration modes correspond to the hexagonal cadmium sulfide (CdS) structure and agree with previous reports [2,31,41]. Furthermore, the intensity of the $A_{1}$ (1LO) peak decreases due to the incorporation of aluminum in the CdS structure, increasing the FWHM and changing the crystallinity of the films. Additionally, we can see that it is possible to identify multiphonon processes under backscattered Raman conditions [1]. Thus, the $A_{1}$ (2LO) overtone was fitted using a GaussAmp function (see Table 2 and Figure 3b–d). In this way, it was possible to observe the fundamental $A_{1}$ (1LO) and its overtones, along with its $A_{1}$ (2LO) Raman scattering replica. Previous reports [31,42] showed band groups for CdS under resonance and non-resonance Raman conditions, as can be seen in Figure 3, confirming that it is possible to determine some vibration modes by means of a data fit. In addition, multiphonon Raman scattering could be favored by improving the crystalline quality of the films. Furthermore, using the ratio I_2LO_/I_1LO_, the strength of the exciton–phonon (EP) coupling could be estimated [43]. Here, the ratios are 1.18 (R = 0.30), 4.84 (R = 0.40), and 4.59 (R = 0.50), respectively. For CdS films, I_2LO_/I_1LO_ will be estimated from the Raman spectrum, which is very close to R = 0.30. It should also be noted that the strength of the EP coupling increases remarkably with the aluminum content from R = 0.35, as observed in the Raman spectra reported by Herrera-Molina et al. [1]. The enhancement of crystallinity with Al doping allows the exciton and phonon to propagate in a wider range and for a longer time, further strengthening the EP couplings [40,43]. The results obtained agree with the reported XRD, AFM, and SEM analyses (see Figure 1 and Figure 2).

**Figure 4:**
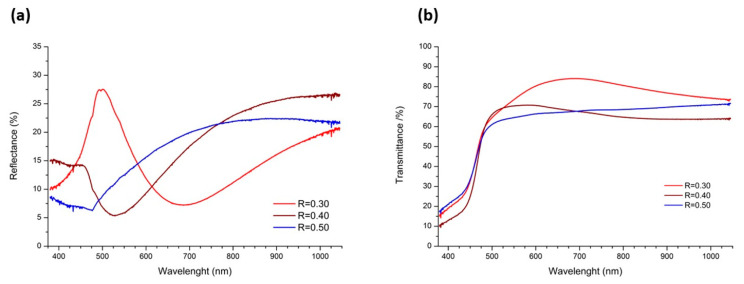
(**a**) Reflectance spectra and (**b**) transmittance spectra of Raman spectra of CdS:Al thin films.

Transmittance and reflectance measurements were performed (Figure 4) for all thin films grown at different [Al^3+^]/[Cd^2+^] ratios (R = 0.30, 0.40, and 0.50) to determine the capacity of the thin films as windows in solar cell applications. It is observed that in the region from 500 to 1000 nm, the sample R = 0.30 exhibits a high transmittance that exceeds 80% and shows a sharp reflectance peak at about 500 nm. On the other hand, for R = 0.40, there is a minimum reflectance at the same wavelength as the maximum transmittance, indicating the good optical quality of this thin film and therefore its potential use in optoelectronic devices. In this sense, these films have good crystallization and structural homogeneity, which minimizes photon scattering caused by defects in the structure [1,4]. This reflection peak is related to the lower amount of surface grains compared to the other doped films in addition to the sharpness of its XRD peak. The higher crystallinity of the sample, together with its smoother surface, leads to a well-defined reflectance peak. Thus, its good optical quality is related to its surface morphology and crystallographic parameters. In addition, the higher transmittance could be explained by the increase in grain size, which could lead to less scattering between the grain boundaries [11]. Therefore, the use of these thin films as window layers in a solar cell can increase the efficiency of the cell and thus increase the generation of electron–hole pairs in the absorber layer.

On the other hand, the thickness, the refractive index (*n*), and the optical energy bandgap (*E_g_^opt^*) of the films were estimated and reported in Table 1. The thickness of the films and their refraction indexes were estimated using the software Filmeasure (Version 8.7, Filmetrics, San Diego, USA)*,* which fits the transmittance and reflectance spectra to a layer model, considering the samples to be constituted of an air/CdS/glass system. An increase in the thickness of the CdS:Al films is observed as the Al content increases due to the presence of grains on their surfaces with sizes of around 200 nm and lower surface roughness, as is shown in Figure 1d–f. However, Figure 2a shows that for the undoped film, the surface is smooth with a grain diameter of approximately 80 nm. In the case of the refraction index (*n*), a decrease in the value from the maximum at R = 0.30 is observed. As the Al-doping increases, it approaches the value reported in Ref. [1] for undoped CdS films.

Additionally, the *E_g_^opt^* of the films was first calculated using the Kubelka–Munk relation, where the *E_g_^opt^* was determined using the intercept between (F(R)*hν*)*^n^* and *hν* [1,44], and second by deriving the absorbance spectrum from *hν* and obtaining the *E_g_^opt^* value with the maximum peak [44,45], which is closer to the exact value. It was observed that the *E_g_^opt^* increases and its crystallite size decreases due to aluminum content in the CdS crystal structure (see Table 1 and Figure S2). Here, we related the *E_g_^opt^* for doped films with the values of D and ε, as reported in Table 1. Furthermore, previous reports [1,26,27,46] confirm that a decrease in the crystallite size increases the strain in the films, and therefore leads to an increase in their *E_g_^opt^*. Here, we found both an increase in the *E_g_^opt^* and ε for doped films.

**Figure 5:**
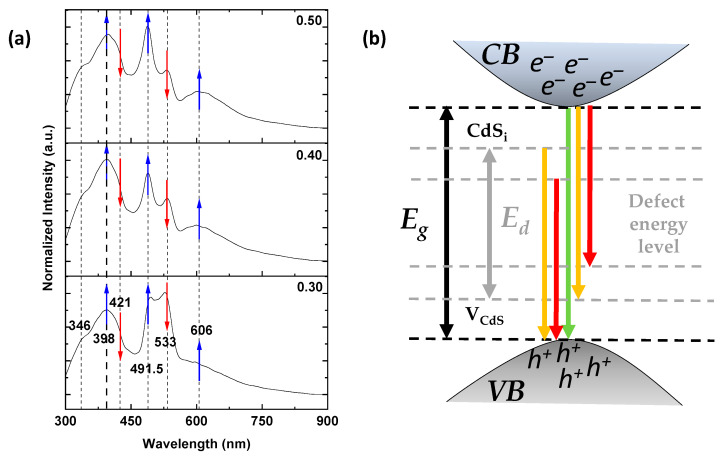
(**a**) Normalized photoluminescence spectra of the thin films showing intensity changes due to Al content (blue and red arrows). (**b**) Schematic energy level diagram showing some of the principal defect levels in CdS:Al thin films.

Figure 5 shows the photoluminescence (PL) spectra of thin films excited at 250 nm. Here, emission peaks in the blue and visible regions were observed with more than one emission peak in these regions attributed to the grain boundaries of the crystalline nature of the doped cadmium sulfide thin films. These emission peaks remain stable with respect to their wavelength but present a change in intensity when increasing the content of aluminum. The photoluminescence spectrum for each thin film exhibits an observable peak at around 533 nm (2.33 eV), which could be assigned to the band-to-band transition and is related to the *E_g_^opt^* of the CdS structure, which agrees well with the absorbance measurements (not shown). The peak observed at around 492 nm (2.53 nm) was previously assigned to the interstitial and vacancy defects in the CdS crystal structure [2]. Furthermore, the emission peak at 492 nm has a low intensity in relation to the emission peak at 533 nm for the undoped film. Additionally, its intensity increases with the Al content (see Figure 5a, blue and red arrows). In addition, this intensity variation of the emission peaks is associated with structural changes such as the increase in grain size (see the AFM and SEM images).

On the other hand, the blue emission peaks are assigned to the donor–acceptor pair, and the broad yellow-red band centered at 606 nm (2.05 eV) is not observed for the undoped film reported by Herrera-Molina et al. [1]. However, for all thin films with Al content, the broad yellow-red band features more than one superimposed emission peak attributed to aluminum, implying that Al incorporation into the CdS structure clearly affects PL’s properties in the films. Moreover, the peak at 606 nm can be attributed to recombination through localized states on the surface and/or radiative and non-radiative processes (green peaks) induced by the aluminum content as reported by Herrera-Molina et al. [1], Kulkarni et al. [41], and Ahmad-Bitar [47], respectively. Figure 5b shows a schematic energy level diagram observed in the PL spectra of Figure 5a for the deposited thin films, where the band-to-band transition and defect energy levels are present, which could be responsible for the photoemission.

## 3. Materials and Methods

### 3.1. Materials 

All chemicals were used without further purification. Thin films were fabricated employing the following reagents: cadmium chloride (20 mL of 0.05 M CdCl_2_ * 2.5 H_2_O, 98%, Sigma-Aldrich, St. Louis, MO, USA), sodium citrate (20 mL of 0.5 M C_6_H_5_O_7_Na_3_, 99%, Sigma-Aldrich), potassium hydroxide (5 mL of 0.5 M KOH, 88 %, J. T. Baker), Thiourea (10 mL of 0.5 M CS(NH_2_)_2_, 99%, Sigma-Aldrich), and 5 mL of pH 10 borate buffer (Sigma Aldrich). In situ doping of CdS films was performed by adding aluminum chloride (AlCl_3_ * 6H_2_O, 99%, Sigma-Aldrich) to the mixture with different molar ratios in the solution, R = [Al^3+^]/[Cd^2+^] = 0.30, 0.40, and 0.50, where the initial concentration of Cd remained constant. 

Note of Toxicity. Cadmium is a highly toxic metal suspected of being carcinogenic, as reported in the chemical safety sheet of the reagent used in the synthesis. Therefore, the synthesis of the films was carried out with the corresponding laboratory safety parameters for the handling of highly complex and toxic reagents.

### 3.2. Sample Preparation

**Figure 6:**
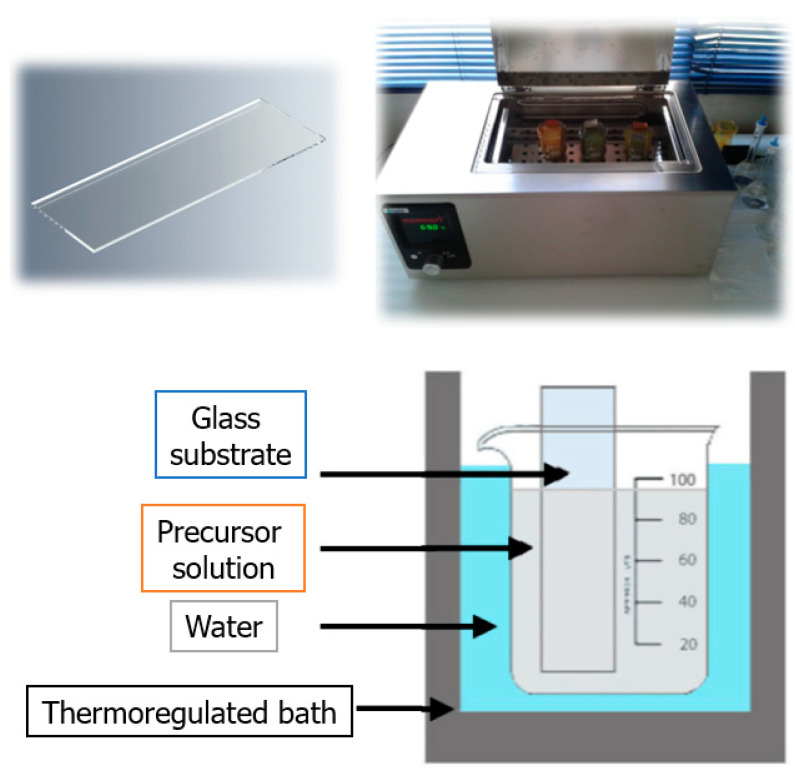
Schematic diagram of the synthesis process used to obtain the thin films. A glass substrate (**top left**) is placed in a thermoregulated bath (**top right**) in a precursor solution at 70 °C for 120 min. The substrate is placed vertically in the solution (**bottom**).

Sample preparation has been previously reported in Ref. [7] and is described below: Thin films of aluminum-doped cadmium sulfide (CdS:Al) were fabricated onto glass substrates using the chemical bath deposition (CBD) method from an alkaline aqueous solution. First, all glass substrates were cleaned in deionized water and 2-propanol using an ultrasound bath for 30 min. After that, the glass substrates were dried with nitrogen (N_2_) gas. In situ doping of CdS films was performed by adding aluminum chloride to the mixture with a constant initial content of cadmium. The total volume of the deposition solution was made equal to 100 mL by the addition of de-ionized water and then the glass substrates were vertically fixed inside the beaker during the reaction in a chemical bath. In this way, Al-doped CdS thin films were grown at a temperature of 70 °C for 120 min. The thin films were air-dried under laboratory conditions. Subsequently, all samples were annealed at 300 °C for 2 h to remove organic impurities from them. The required amounts of reagents are described in Section 3.1. The schematic procedure used for the synthesis of the thin films is illustrated in Figure 6.

### 3.3. Characterization Techniques

#### 3.3.1. X-ray Diffraction (XRD) and AFM Studies 

The crystal structure and morphology of the synthesized thin films were characterized using an X-ray diffractometer from PANnalytical with Bragg–Brentano geometry (X´pert Pro diffractometer, 45 kV/40 mA, using Cu-K_α1/α2_ radiation (*λ* = 0.1540598 nm/0.1544426 nm)) and NaioAFM Nanosurf in contact mode. The XRD patterns were recorded at room temperature over the angular range of 2θ = 20–70° with a step size of 0.02°. Additionally, SEM micrographs were obtained using a Hitachi scanning electron microscope SU3500 at 20 kV and a chamber pressure of 80 Pa.

#### 3.3.2. Spectroscopy Studies

A DXR micro-Raman spectrometer was used for a backscattering geometry of the sample from ThermoFisher Scientific with a diode laser of 532 nm (2.33 eV). Ultraviolet-visible (UV-vis) absorption measurements of the thin films were recorded using a single monochromator V-770 UV-vis-NIR spectrophotometer from Jasco in the wavelength range of 200–800 nm. Room temperature photoluminescence (RT-PL) measurements were performed with a Perkin Elmer Spectrofluorometer LS55 (Xenon source).

## 4. Conclusions

The influence of the metallic ion Al^3+^ in the structural, morphological, vibrational, and optical properties of CdS thin films was studied. All the films show a hexagonal CdS structure, and no other phases were observed. Structural parameters such as crystallite size and strain have been determined. The crystallite size decreased and the strain increased due to the incorporation of Al^3+^ ions that replace the Cd^2+^ ions in the structure. AFM and SEM results show large grains with a diameter between 200 and 400 nm in doped films. Raman scattering confirms that the h-CdS structure of the films and the intensity of the LO modes change with Al content. Furthermore, Al doping in CdS could favor the multiphonon Raman scattering as well as the exciton–phonon (EP) coupling strength by improving the crystalline quality of the films. Measurements of the optical properties indicate that the deposited thin films have a direct energy gap with *E_g_^opt^* values ranging from 2.3 to 2.7 eV. This characteristic is related to the increase in the lattice strain of the films with higher Al content. This Al content also affects the PL properties of the films. The present work shows that Al-doped CdS thin films have potential uses in optoelectronic devices such as solar cells and UV photodetectors due to their good crystallinity and high optical transmittance.

## Figures and Tables

**Table 1:** Crystallite size, thickness, strain, refraction index, and energy gap values of the thin films.

Thin Film R = [Al]/[Cd]	Crystallite Size D ± 1 (nm)	Thickness (nm)	Strain ε ± 0.8 (× 10^−4^)	Refraction Index N	Optical Energy Bandgap E_g_^opt^ (eV)	
					K-M	1st Derivative
0.30	12	173	31.3	2.0(7)	2.5(6)	2.6(4)
0.40	11	213	40.5	1.7(8)	2.5(8)	2.6(7)
0.50	14	193	29.7	1.4(4)	2.5(5)	2.6(5)

**Table 2:** Vibration modes found in the Raman spectra of fabricated thin films by the CBD method.

Raman Shift, *v*_exp_ (cm^– 1^)						Symmetry
Ref. [1]	Ref. [36]	Ref. [37]	R = 0.30	0.40	0.50	
304	301	306–308	302.8	303.7	302.8	$A_{1}$ (1LO)
473.7	470	-	485.5	477	458	$A_{1}$ (2LO) multiphonon Raman scattering
564	-	556–560	561.8	571.1	563	
-	-	-	599.2	-	602.7	
620.3	640	-	630.5	640	618	
685.7	670	-	688.3	-	635.5	

- indicating that the vibrational mode was not reported or detected in the measurement.

## Data Availability

The CdS dataset used and analyzed during the current study is available by mutual agreement between the corresponding author and Elsevier and consists of license details and the terms and conditions provided by Elsevier and the Copyright Clearance Center. License number 5494560381898.

## Supplementary Materials

The following supporting information can be downloaded at: https://www.mdpi.com/article/10.3390/molecules28083626/s1. Figure S1: XRD pattern of thin films grown at different [Al]/[Cd] ratios; Figure S2: Crystallite size and energy gap versus [A]/[Cd] ratio for the thin films.
