# Supplementary material for: Study of the Properties of CdS:Al (R = [Al3+]/[Cd2+] = 0.30, 0.40, 0.50) Thin Films Grown by the CBD Method in an Ammonia-Free System
Source: Molecules. 2023 Apr 21;28(8):3626. doi: 10.3390/molecules28083626 (PMC10146886; doi:10.3390/molecules28083626)
Supplement: Supplementary file 1 [file molecules-28-03626-s001.zip › molecules-2270510-supplementary.pdf]

Article

Study of the properties of CdS:Al (R = [Al]/[Cd] = 0.30, 0.40, 0.50) thin films grown by the CBD method in an ammonia-free system

R. Prasanna-Kumari [1], Daniela Herrera-Molina [1], Arturo Fernández-Pérez [2], Jesús E. Diosa [1,3], Edgar Mosquera-Vargas [1, 3,*] ,

[1] Grupo de Transiciones de Fase y Materiales Funcionales, Departamento de Física, Universidad del Valle, Santiago de Cali 760032, Colombia; prasanna.raju@correounivalle.edu.co , daniela.herrera.molina@correounivalle.edu.co

[2] Departamento de Física, Facultad de Ciencias, Universidad del Bio-Bio, Collao 1202, Concepción 4030000, Chile; arturofe@ubiobio.cl

[3] Centro de Excelencia en Nuevos Materiales (CENM), Universidad del Valle, Santiago de Cali 760032, Colombia; jesus.diosa@correounivalle.edu.co ,

* Correspondence: edgar.mosquera@correounivalle.edu.co (EM-V)

Supporting information

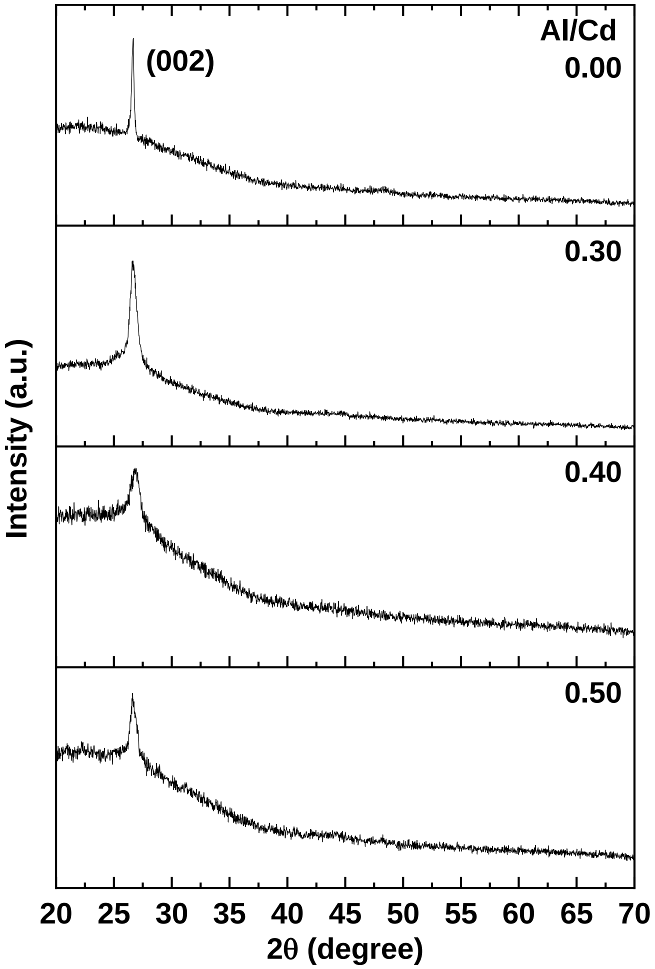

Figure S1. XRD pattern of thin films grown at different [Al]/[Cd] ratios. From Ref. [1].

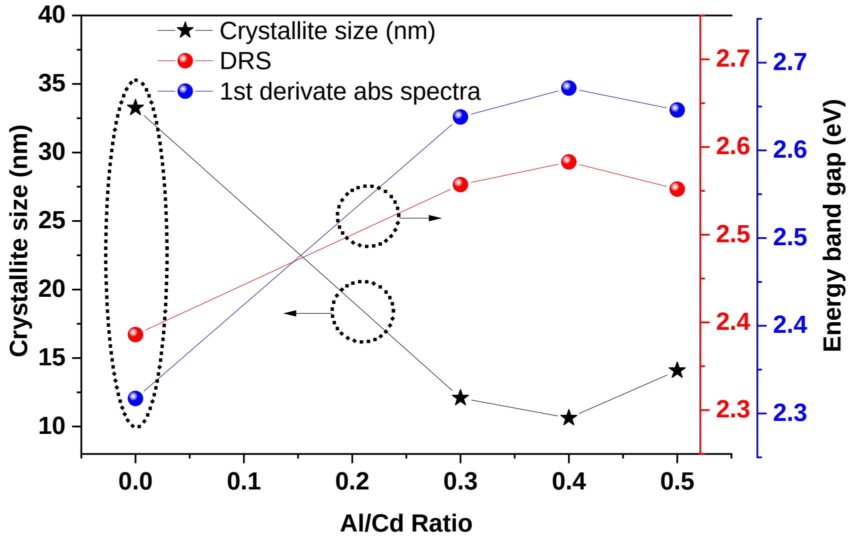

Figure S2 Crystallite size and energy gap versus [A]/[Cd] ratio for the thin films. From Ref. [1].
